# Evaluation of pelvic asymmetry and lower limb functional shortening in a cohort of children re-examined after a ten-year observation

**DOI:** 10.1186/1748-7161-9-S1-O1

**Published:** 2014-12-04

**Authors:** Anna Kluszczynska, Adam Kluszczynski, Jan Raczkowski, Piotr Siwik

**Affiliations:** 1 Troniny Children Rehabilitation Center, Częstochowa, Poland; 2 Medical Sciences University, Lodz, Poland; 3 Medical Sciences University of Varmia and Masuria, Olsztyn, Poland

## Background

The study is a cohort study of children for the occurrence of pelvic asymmetry and functional lower limb shortening.

## Aim

The aim of the study was to assess changes in the prevalence of symmetry of the pelvis and lower limb functional length in children and adolescents after a 10 year observation.

## Material and methods

A group of 100 children and adolescents, aged 4-16 years, including 58 girls and 42 boys, were examined initially in 1997 and then re-examined 10 years later. Clinical examination was performed by the same observer (first author), using the same methodology. The exam consisted of: (1) clinical assessment of pelvic symmetry, (2) functional lower limb discrepancy assessment based on Rippstein plurimeter measurement of iliac spines position. Squared Chi test was used for comparison.

## Results

The age at follow-up ranged from 14 to 26 years. Clinical pelvis asymmetry was identified in 23.8% and 71.4% of boys during the first and second exam, respectively and in 22.4% and 46.5 % of girls, respectively. In boys, the functional lower limb discrepancy was found in 9.7% and 18.6% during the first and the second examination, respectively, with prevalence in respect to the left leg shortening 2.4% and the right leg 37.8%, respectively, difference significant. In girls, in the first study, the functional shortening of the lower limb was found more frequently in the left leg 25.4% compared to 8.5% in the right. In the second study, the functional shortening of the left lower limb was found in 18.7% in 28.9% for the right one.

## Conclusions

1. The second examination revealed a significant increase in occurrence of pelvic asymmetry.

2. After 10 years the functional shortening of one of the lower limbs increased in both sexes.

3. In the first study, a functional shortening of the left lower extremity predominated in the girls, while in the second study it was the right lower extremity and was identified in the boys.

